# Coverage probability analysis of three uplink power control schemes: Stochastic geometry approach

**DOI:** 10.1186/s13638-018-1120-7

**Published:** 2018-06-07

**Authors:** Prasanna Herath, Chintha Tellambura, Witold A. Krzymień

**Affiliations:** Department of Electrical and Computer Engineering, Donadeo Innovation Centre for Engineering, University of Alberta, Edmonton, T6G 1H9 AB Canada

**Keywords:** Cellular networks, Coverage probability, Fractional power control, Stochastic geometry layout, Uplink

## Abstract

In cellular networks, each mobile station adjusts its power level under control of its base station, i.e., through uplink transmit power control, which is essential to reach desired signal-to-interference-plus-noise ratio (SINR) at the base station and to limit inter-cell interference. The optimal levels of transmit power in a network depend on path loss, shadowing, and multipath fading, as well as the network configuration. However, since path loss is distance dependent and the cell association distances are correlated due to the cell association policies, the performance analysis of the uplink transmit power control is very complicated. Consequently, the impact of a specific power control algorithm on network performance is hard to quantify. In this paper, we analyze three uplink transmit power control schemes. We assume the standard power-law path loss and composite Rayleigh-lognormal fading. Using stochastic geometry tools, we derive the cumulative distribution function and the probability density function of the uplink transmit power and the resulting network coverage probability. It is shown that the coverage is highly dependent on the severity of shadowing, the power control scheme, and its parameters, but invariant of the density of deployment of base stations when the shadowing is mild and power control is fractional. At low SINRs, compensation of both path loss and shadowing improves the coverage. However, at high SINRs, compensating for path loss only improves coverage. Increase in the severity of shadowing significantly reduces the coverage.

## Introduction

Both uplink and downlink transmit power control (TPC) is an integral part of modern cellular system standards (e.g., Long-Term Evolution (LTE), LTE-advanced) to control the transmit power of mobile stations (MSs) and base stations (BSs), respectively, in order to mitigate inter-cell and intra-cell interference, while achieving energy savings, improving connectivity, and maintaining a required signal-to-interference-plus-noise ratio (SINR) [1]. Uplink power control is essential to the operation of CDMA cellular systems (e.g., the 3G cellular systems). Without the uplink power control, these systems would simply not work, due to the near-far effect on their uplink [2]. The simplest uplink TPC is to ensure that all user transmissions reach the same SINR at the base station (BS), which however requires that those encountering high path loss transmit with much higher power. In contrast, fractional power control, standardized by 3rd Generation Partnership Project (3GPP), compensates for the path loss between the MS and its serving BS, and higher path loss users (e.g., cell-edge users) are allowed to operate at a lower SINR, thus reducing inter-cell interference. TPC (both downlink and uplink) is especially important for dense heterogeneous cellular networks (HCNs), the layouts of which may be very irregular. In HCNs, uplink interference from a neighboring cell can be very strong [3], and battery-powered MS handsets need to save energy. All these reasons have motivated the development of various uplink TPC schemes to improve the total network throughput, cell-edge user performance, and energy efficiency [3–16].

Therefore, it is critically important to understand and quantify the performance of both uplink and downlink TPC schemes. Fortunately, the downlink has been widely modeled and optimized using stochastic geometry [3, 5, 17–21]. Stochastic geometry facilitates tractable analysis of HCNs, where the locations of the BSs, APs (access points), and MSs are distributed according to mathematically tractable point processes [17, 20, 22]. For example, the homogeneous Poisson point process (PPP) has been widely used to study HCNs [17, 20, 22]. However, the uplink studies have been relatively limited but have become necessary due to, for example, applications such as cloud processing and storage [23].

Location-dependent power control and orthogonal multiple access make uplink analysis more challenging. To elaborate, there is only one randomly located MS per cell per resource block due to the use of orthogonal frequency division multiple access (OFDMA). Thus, cell association policy couples the locations of MSs. Consequently, even when all MSs in the network form a PPP, the set of active MSs in a given resource block does not form a PPP. Moreover, the transmit powers of MSs, which typically depend on the MS-BS distances and random channel gains due to shadowing and multipath fading, are highly variable and correlated. This is because, although MS-BS distances of different cells are identically distributed, they are not statistically independent. All these conditions pose fundamental challenges for the analysis of uplink TPC performance.

In previous research, the aforementioned challenges have been overcome in two ways. First, one neglects or only partially captures the dependency among locations of interfering MSs and simply assumes them to form a homogeneous/inhomogeneous PPP. Therefore, we call this the PPP-approximated MS model. The correlation between the tagged MS and the interfering MSs is captured by considering an appropriate interference protection region around the serving BS. In that model, the MS transmit powers are also assumed to be independent and identically distributed (i.i.d.). The exact distribution depends on the power control scheme adopted. Second, one considers a fully loaded network, i.e., each cell has an active uplink transmission scheduled per time-frequency resource block, and MSs form a homogeneous PPP [24]. When the spatial dependency between co-channel MSs is neglected, they are assumed to form a homogeneous PPP. This makes the density of MSs per resource block equal to the density of BSs in the network. Each MS is assumed to be associated with the BS that provides the highest area averaged received signal power. To capture the correlations among MS-BS distances, the associated BS of each MS is assumed to be uniformly distributed in the Voronoi cell of the MS. We refer to this approach as the downlink equivalent model [5].

### Prior related research

In general, uplink power control may be a combination of two mechanisms: open-loop and closed-loop [1]. The open-loop power control mechanism adjusts MS transmit power according to downlink path loss estimates, while the closed-loop one involves adjusting the MS transmit power according to power control signals sent by the BS on the downlink that are determined by BS estimates of the received uplink signal power [1]. Mathematical analysis of closed-loop power control has so far been intractable. Hence, this work considers only open-loop power control.

For modern OFDMA-based or similar cellular networks, open-loop TPC (TPC for simplicity) attempts full/fractional compensation of path loss and/or shadowing [5, 25]. Common schemes are path loss inversion (PLI) and path loss and shadowing inversion (PLSI). In the following, we summarize recent uplink TPC studies and highlight their contributions.

PLI, which compensates for path loss only, is considered in [3, 5–9, 14–16, 24, 26]. The downlink equivalent model is used by [5, 24], while the PPP-approximated MS model is adopted by [3, 6–9, 14–16, 26]. Reference [5] has introduced the downlink equivalent model and has derived the coverage and average rate of uplink fractional PLI power control in a single-tier network. Inter-cell interference mitigation through uplink fractional frequency reuse and fractional PLI power control is investigated in [24]. For a multi-tier HCN, [8] proposes a tractable and general model to characterize the uplink signal-to-interference ratio (SIR) and rate distribution. The first uplink analysis with multiple antenna BSs is presented in [9] considering a generalized version of fractional PLI power control. The coverage probability and achievable rate are derived for maximum ratio combining and optimum combining at the BS. All works thus far consider fractional PLI. In contrast, full PLI, which maintains a constant area mean (after averaging over variations due to shadowing and multipath fading [2]) received power at BSs, is considered in [3, 7, 26]. The outage probability and spectral efficiency of both single-tier and multi-tier cellular networks are investigated in [3]. Symbol error rate analysis of multi-tier HCNs is presented in [7]. A two-tier HCN consisting of femtocells and macrocells is considered in [26]; upper and lower bounds for the outages of femtocell and macro users are derived there. SIR distribution in a two-tier HCN is investigated in [6], where the MS transmit power is selected out of a finite set of discrete values to maintain the area mean received power above or equal to a predefined target value. Reference [14] investigates uplink outage probability in a multi-channel environment and captures the load variation on BSs. Uplink SINR and rate distribution in a massive multiple input multiple output (MIMO) network are investigated in [16].

In all previously mentioned references, uplink performance is investigated by averaging over the respective underlying point processes considered for the spatial distribution of users and BSs. Recently, [27, 28] have investigated the uplink coverage probability of arbitrary, but fixed, realization (meta distribution of SIR) of Poisson cellular networks. Both these references consider fractional PLI power control.

References [4, 10–12, 15] consider PLSI power control (i.e., compensation of both path loss and shadowing). The downlink equivalent model is adopted by [4], but PPP-approximated MS model is used by [10–12, 15]. Reference [10] considers an interference aware PLSI scheme, where interference from each MS to the most interfered BS is kept under a predefined value. Reference [11] evaluates the uplink interference in a two-tier HCN considering multi-type users and BSs. Uplink capacity in a two-tier direct sequence CDMA (DS-CDMA) HCN consisting of macro BSs and femto APs is considered in [12]. Our work [4] investigates coverage probability of a single-tier cellular network in composite Rayleigh-lognormal fading channels, where we consider fractional inversion of path loss and complete compensation of shadowing. Uplink SINR and rate distribution in a multi-tier cellular network are investigated in [15]. Joint uplink and downlink rate coverage (joint probability of uplink and downlink rate/SINR exceeding their respective thresholds) is also investigated there.

### Motivation and our contribution

Our main goal is to study and characterize the impact of power control and shadowing on the uplink performance. The effectiveness of shadowing compensation by power control is also in the focus. Shadowing may degrade the performance of modern HCNs that include both macro and low-power, small form-factor small cells, which have low antennas. In such environments, a significant impact of shadowing is to be expected. Shadowing specifically refers to variations in the signal strength over distances proportional to the length of an obstructing object (10–100 meters) in outdoor environments and less in indoor environments [29]. Experiments have confirmed that typical shadowing effects can be modeled as log-normal [2, 29] and that distribution has thus been widely used to study the effects of shadowing [2, 29–31]. Overall, power control schemes that alleviate shadowing in addition to path loss (i.e., PLSI) will undoubtedly have a beneficial effect on the uplink coverage, data rates, and power efficiency.

Although uplink PLSI studies exist [10–12], their system models appear to be not flexible enough to investigate the effect of uplink TPC in modern cellular networks. For example, [10, 11] consider shadowing as a random displacement on the MS point process. This prevents the consideration of different shadowing levels for different radio channels, desired and interfering channels for example. Reference [12] investigates the cellular uplink with PLSI power control. However, it investigates a two-tier DS-CDMA cellular network. Therefore, the results of this research are not fully applicable for modern OFDM or discrete Fourier transform spread OFDMA (DFT-s-OFDM)-based cellular uplink. With this motivation, in our previous work [4], we investigated fractional compensation of path loss and complete inversion of shadowing (number 3 in the list below) in an OFDMA/DFT-s-OFDM-based cellular network considering more practical composite Rayleigh-lognormal fading channels. In this paper, we extend our study by considering additional two power control schemes and comparing the performance of these schemes under various network configurations and propagation conditions.

The three TPC schemes provide full and/or fractional compensation for

1. Path loss only
2. The aggregate effect of path loss and shadowing
3. Fractional compensation of path loss and complete inversion of shadowing

With these, the transmit power of an MS becomes a random variable, whose statistics depend on the MS-BS distance and shadowing. We thus use the downlink equivalent model of [5] and the PPP [32] to characterize the spatial distribution of MSs. We derive the cumulative distribution function (CDF) and the probability density function (PDF) of the transmit MS power. Analytically derived expressions are validated via simulations. These expressions help us to quantify the impact of power control and shadowing on the coverage probability. Specifically, the main contributions of this paper can be summarized as follows:

- We provide analytical expressions for PDF and CDF of the transmit power and coverage probability for the three power control schemes considering composite Rayleigh-lognormal fading and path loss. To evaluate the complicated integrals, we use the Gauss-Hermite and Gauss-Laguerre quadratures to express them as weighted sums of function evaluations. This provides a powerful, flexible platform to evaluate the effects of different shadowing levels. Computing the Gauss quadrature nodes and weights is very simple with Golub-Welsch (GW) algorithm [33], which utilizes the eigenvalues and eigenvectors of the symmetric tridiagonal matrix formed by the recurrence relations to compute the nodes and weights.
- We investigate in detail the effects of shadowing, power control factor, and BSs’ density on the coverage probability using analysis and simulations. We show that under all three power control schemes, density of BSs has no significant effect on the coverage.
- Comparing the performance of three power control schemes, we show that at low SINRs (cell edge users), compensating for both path loss and shadowing improves the coverage probability. However, at high SINRs (users closer to BS), compensating for path loss only is more effective.

**Notation:** ||*x*−*y*|| is the Euclidean distance between $ x,y \in \mathbb {R}^{2}$. The probability of event *A* is Pr(*A*). $\mathcal {E}\left [\cdot \right ]$ is the expectation operation. *f*_*X*_(·) and *F*_*X*_(·) represent the PDF and CDF of a random variable *X*. The Laplace transform of the PDF of a random variable *X* is denoted as $\mathcal {L}_{X}(s) = \mathbb {E}_{X}\left [e^{-sX}\right ]$. ${}_{2}F_{1}\left (\alpha, \beta ; \gamma ; z\right) = \frac {1}{B(\beta, \gamma - \beta)}\int _{0}^{1} t^{\beta -1}(1-t)^{\gamma -\beta -1}(1-tz)^{-\alpha } dt,\ \text {Re}\ \gamma > \text {Re}\ \beta > 0$ ([34], eqs. 9.14.2, 9.111) is the hypergeometric function, where $B(x, y) = \int _{0}^{1} t^{x-1} (1-t)^{y-1}dt $ ([34], eq. 8.380.1) is the beta function.

## System model, power control, and assumptions

This section presents the network setup, three open-loop power control schemes considered, and key assumptions needed for tractable mathematical analysis.

### System model

This research considers uplink transmission in a cellular network with the following network configurations and assumptions. We use the downlink equivalent model proposed in [5], which is described by the following six points.

1. Network consists of a single class of BSs with density *λ*>0. Similar to [3, 5], we consider minimum path loss-based association policy. Therefore, each MS is associated with the BS providing the highest area mean received power [2]. This is equivalent to connecting to the closest BS. Considering minimum path loss association is motivated by two reasons. First, investigating the effectiveness of compensating for shadowing as a part of power control is one of the aims of this paper. Therefore, we refrain from considering shadowing and fast fading for the cell association. Second, this association policy is simple and also avoids frequent handoffs [35]. An orthogonal multiple access technique is used, for example, OFDMA or DFT-precoded OFDMA (single-carrier FDMA).
2. Universal frequency reuse [2], which allows every cell in the network to reuse the same set of carrier frequencies. The network is fully loaded, i.e., each BS has an active uplink transmission scheduled for each time-frequency resource block.
3. The locations of MSs operating in a particular time-frequency channel form a homogeneous PPP *Φ*. Due to orthogonal channel assignment and the assumption of a fully loaded network, *Φ*={*x*_1_,*x*_2_,…}, where $x_{k} \in \mathbb {R}^{2}$ has the intensity *λ*.
4. With this network setup, each BS is uniformly distributed in the Voronoi cell of its corresponding MS [5, 24]. This is referred to as the downlink equivalent model for uplink communication.
5. All the radio channels are subject to power-law path loss and composite Rayleigh-lognormal fading. Although the severity of shadowing depends on the density and locations of shadowing objects, considering different shadowing levels for each cell is mathematically intractable. Therefore, we consider only two values for the standard deviation of the shadowing process: *σ* for the local environment (locality of the BS serving MS *z*_0_) and *ξ* for interfering cells. Channel power gains due to small-scale fading are assumed to be independently distributed across all MS-BS pairs.
6. We investigate the coverage probability of a randomly chosen MS *z*_0_∈*Φ*. Since a homogeneous PPP in $\mathbb {R}^{2}$ is translation and rotation invariant, without loss of generality, the BS associated with *z*_0_ is assumed to be located at the center of the network. The network is assumed to be interference-limited; therefore, the background thermal noise is ignored.

Reference [5] has investigated the accuracy of this model and has shown that it accurately models the uplink transmissions in a cellular network.

### Power control schemes

We consider three fractional power control schemes, which compensate for path loss and shadowing. For a selected power control scheme, we assume all the MSs use the same set of power control parameters.

#### **Scheme 1**

Fractional/full path loss compensation.

This scheme aims to compensate for the effect of path loss on the received signal power. Therefore, the transmit power *P*_*z*_ of MS *z*∈*Φ* associated with the BS y^1^ is given by

1 $$  P_{z} =\rho \left(l(z,y)\right)^{-\eta},  $$

where *ρ* is a constant and *l*(*z,y*) is the channel power gain due to path loss. For power-law path loss model, *l*(*z,y*)=||*z*−*y*||^−*α*^, where *α*>2 is the path loss exponent. The power control factor is denoted by *η*∈[0,1]. *η* can be interpreted as a fairness parameter, where higher value helps the cell edge users meet their SIR target but at the cost of increasing the interference level in the network [15]. This can reduce the SINR experienced by cell center users. *η*=1 represents complete elimination of path loss while *η*=0 represents no power control (*ρ* is a constant, value is the same for all the MSs).

#### **Scheme 2**

Fractional/full compensation for both path loss and shadowing.

With this power control, the transmit power of MS *z*∈*Φ* is given by

2 $$  P_{z} = \rho \left(l(z,y)h_{zy}\right)^{-\eta},  $$

where *h*_*zy*_ is the shadowing power gain. Therefore, *η*=1 (*η*=0) represents complete compensation for path loss and shadowing (no power control).

#### **Scheme 3**

Fractional compensation for path loss and complete inversion of shadowing.

This scheme completely eliminates the shadowing effect but only partially inverts path loss. Therefore, the transmit power of MS *z*∈*Φ* is given by

3 $$  P_{z} = \rho \left(l(z,y)\right)^{-\eta} h_{zy}^{-1}.  $$

In (3), power control factor *η*∈[0,1] determines the degree of path loss compensation. That is, *η*=1 (*η*=0) gives a complete inversion of path loss (path loss is not compensated for and only shadowing is inverted).

### Downlink equivalent model

As mentioned before, correlations among transmit powers and the locations complicate the analysis. To overcome this, we will use the downlink equivalent model [5] and make two additional assumptions to obtain the PDF of the transmit power for each TPC scheme.

#### **Assumption 1**

According to the system model described earlier, the BS is uniformly distributed in the Voronoi cell of the MS being served. By replacing the Voronoi cell with $\mathbb {R}^{2}$, the distance between an MS and its associated BS follows the Rayleigh PDF given by

4 $$\begin{array}{*{20}l} f_{r}(r) &= 2 \pi \lambda r\ {exp}\left(-\pi \lambda r^{2}\right), \ 0 < r < \infty. \end{array} $$

This assumption is also made in [5], and it is shown that the loss of accuracy due to this assumption is minimal. We will use it to derive the PDFs of the MS transmit powers.

#### **Assumption 2**

All MS-BS distances are i.i.d. with PDF (4).

#### **Assumption 3**

Transmit powers of different MSs are also i.i.d.

References [3,5] showed that the dependencies between MS-BS distances and transmit powers are in fact weak, and above assumptions yield accurate results. In Section 5, we also test the validity of these assumptions by comparing numerical and simulation results and show that the loss of accuracy due to these assumptions is negligible.

## Transmit power analysis

Here, we derive the transmit power PDFs and CDFs.

### Scheme 1: Fractional compensation for path loss

In this scheme, each MS adjusts its transmit power according to (1). Using Assumptions 1 and 2 and (4), approximate expressions for the CDF and PDF of the transmit power at MSs *z*∈*Φ*, *P*_*z*_, can be derived as given in the following lemma.

#### **Lemma 1**

In a single-tier Poisson cellular network with the closest BS cell association and fractional path loss inversion power control, the CDF and the PDF of the transmit power *P*_*z*_ are given by

5 $$\begin{array}{*{20}l}  F_{P_{z}}(t) &= 1-\text{exp}\left(-\pi \lambda \rho^{-\frac{2}{\alpha \eta}} t^{\frac{2}{\alpha \eta}}\right),\quad 0 < t < \infty. \end{array} $$

6 $$\begin{array}{*{20}l}  f_{P_{z}}(t) = \! \frac{2 \pi \lambda}{ \alpha \eta \rho^{\frac{2}{\alpha \eta}}} \text{exp}\left(-\pi \lambda \rho^{-\frac{2}{\alpha \eta}} t^{\frac{2}{\alpha \eta}}\right) t^{\frac{2}{\alpha \eta}-1}\ \, \\ 0 < t < \infty. \end{array} $$

#### *Proof*

Using (1), the CDF of *P*_*z*_ can be written as

7 $$\begin{array}{*{20}l}  F_{P_{z}}(t) &= \text{Pr}\left[\rho r^{\alpha \eta} < t\right] = \text{Pr}\left[r<\left(\frac{t}{\rho}\right)^{\frac{1}{\alpha \eta}}\right]. \end{array} $$

Substituting the PDF of *r* given by (4) in (7), we obtain the CDF of *P*_*z*_ given in (5). By differentiating (5), we obtain the PDF of *P*_*z*_ given in (6). □

### Scheme 2: Fractional compensation for the aggregate effect of path loss and shadowing

For partial inversion of the path loss and shadowing, the transmit power at the MS *z*∈*Φ*, *P*_*z*_, is given by (2). The CDF and PDF of the transmit power are given below.

#### **Lemma 2**

In a single-tier Poisson cellular network with the closest BS cell association and fractional path loss and shadow inversion power control, the CDF of *P*_*z*_ is given by

8 $$\begin{array}{*{20}l}  F_{P_{z}}(t) &= 1-\sum_{k=1}^{N}\frac{w_{k}}{\sqrt{\pi}}\text{exp}\bigg(-\pi \lambda \left(\frac{t}{\rho}\right)^{\frac{2}{\alpha \eta}} \\ &\quad \times \text{exp}\bigg(\frac{2\sqrt{2} \xi u_{k}}{\alpha}\bigg)\bigg) + O_{N},\ 0<t<\infty. \end{array} $$

The PDF of the transmit power is given by

9 $$ {}\begin{aligned} f_{P_{z}}(t) &= \frac{2 \sqrt{\pi}\lambda}{\alpha \eta \rho^{\frac{2}{\alpha \eta}}}\sum\limits_{k=1}^{N}w_{k} t^{\frac{2}{\alpha \eta}-1} \\ &\quad \times \text{exp}\left(\frac{2\sqrt{2} \xi u_{k}}{\alpha} -\pi \lambda \left(\frac{t}{\rho}\right)^{\frac{2}{\alpha\eta}} \text{exp}\left(\frac{2\sqrt{2} \xi u_{k}}{\alpha}\right)\right) \\ &\quad + \epsilon_{N},\ 0 < t < \infty. \end{aligned}  $$

Here, *N*>1 is an integer which determines the accuracy of the approximation. *O*_*N*_ and *ε*_*N*_ represent the error terms that decrease to zero as *N* increases to infinity. *w*_*k*_ and *u*_*k*_ are weights and abscissas for Gauss-Hermite quadrature of order *N*. For different values of *N*, *w*_*k*_ and *u*_*k*_ are available in [36], Table (25.10), or can be calculated by a simple MATLAB®; program.

*Proof:* See Section 7.1.

### Scheme 3: Fractional compensation for path loss and complete inversion of shadowing

The transmit power of each MS *z*∈*Φ*, *P*_*z*_, is given by (3). The CDF and PDF are given by the following lemma.

#### **Lemma 3**

In a single-tier Poisson cellular network with the closest BS cell association and fractional path loss compensation and complete shadow inversion power control, the CDF is given by

10 $$\begin{array}{*{20}l}  F_{P_{z}(t)} &= 1 - \sum\limits_{k=1}^{N}\frac{w_{k}}{\sqrt{\pi}}\text{exp}\left(-\pi \lambda\left(\frac{t}{\rho}\right)^{\frac{2}{\alpha\eta}}\right. \end{array} $$

11 $$\begin{array}{*{20}l} &\quad \left.\times \text{exp}\left(\frac{2\sqrt{2}\xi u_{k}}{\alpha\eta}\right)\right) + O_{N}, \ \ \ 0 < t < \infty. \end{array} $$

The PDF of the transmit power *P*_*z*_ is given by

12 $$ \begin{aligned} f_{P_{z}}(t) &= \frac{2 \sqrt{\pi} \lambda}{\alpha \eta \rho^{2/\alpha\eta}}\sum_{k=1}^{N} w_{k} t^{\frac{2}{\alpha\eta}-1}\\ &\quad \times \text{exp}\left(\frac{2\sqrt{2}\xi u_{k}}{ \alpha\eta}-\pi \lambda \left(\frac{t}{\rho}\right)^{\frac{2}{\alpha\eta}} {exp}\left(\frac{2\sqrt{2}\xi u_{k}}{ \alpha\eta}\right)\right) \\ &\quad + \epsilon_{N}, \ \ \ 0 < t < \infty. \end{aligned}  $$

Similar to in Lemma 1, *w*_*k*_ and *u*_*k*_ are the weights and abscissas for Gauss-Hermite quadrature of order *N*. *O*_*N*_ and *ε*_*N*_ are the error terms.

*Proof:* See Section 7.2.

## Coverage probability analysis

Next, we derive the coverage probability of the network for the three power control schemes (Section 2.1). Similar to [19], the coverage probability is defined as the probability that a randomly chosen MS *z*_0_∈*Φ* achieves the uplink SIR target of *T*.

### Scheme 1: Fractional compensation for path loss

Under this power control scheme, the SIR at the BS serving MS *z*_0_∈*Φ* can be written as

13 $$\begin{array}{*{20}l}  \text{SIR} = \frac{\rho r_{z_{0}}^{\alpha\left(\eta -1\right)} h_{z_{0}}}{\sum_{z\in \Phi\backslash z_{0}} P_{z} r_{z}^{-\alpha} h_{z}}, \end{array} $$

where $r_{z_{0}}$ is the distance between MS *z*_0_ and its associated BS at the origin. The channel power gain due to composite Rayleigh-lognormal fading is given by $h_{z_{0}}$. The set *Φ*∖*z*_0_ represents all the active co-channel interfering MSs (all MSs of *Φ* except *z*_0_). For MS *z*∈*Φ*, where *z*≠*z*_0_, *r*_*z*_=||*z*|| and *h*_*z*_ are the Euclidean distance and channel gain (Rayleigh lognormal) to the BS. The coverage probability is given by the following theorem.

#### **Theorem 1**

The uplink coverage probability of an MS in a single-tier cellular network under fractional path loss inversion power control is

14 $$\begin{array}{*{20}l}  P_{c} (T) &= 2 \sqrt{\pi} \lambda \sum_{i = 1}^{L} \zeta_{i} \int_{0}^{\infty} r_{z_{0}} \text{exp}\left(- \pi \lambda r_{z_{0}}^{2} \right) \\ &\quad \times \mathcal{L}_{I_{\Phi\backslash z_{0}}}\left(s = \frac{T\ \text{exp}\left(-\sqrt{2} \sigma v_{i}\right)}{\rho r_{z_{0}}^{\alpha (\eta-1)}}\right) dr_{z_{0}} + \epsilon_{L}, \end{array} $$

where

15 $$ \begin{aligned} \mathcal{L}_{I_{\Phi\backslash z_{0}}}(s) &= \text{exp}\left(\frac{-2 \pi^{\frac{1 - \alpha \eta}{2}} \lambda^{\frac{2 - \alpha \eta}{2}} s \rho \ r_{z_{0}}^{2 - \alpha}}{\alpha -2}\right.\\ &\quad \times\sum_{j=1}^{M} \kappa_{j}~\text{exp}\left(\sqrt{2}\sigma x_{j}\right)\! \sum_{q = 1}^{Q} \!\beta_{q} \\ &\quad \left.\times \, \, {\!~\!}_{2}F_{1}\left(1, \frac{\alpha\! -\! 2}{\alpha}, 2\! -\! \frac{2}{\alpha}, \frac{- s \rho~ \text{exp}\left(\sqrt{2}\sigma x_{j}\right) \delta_{q}^{\frac{\alpha \eta}{2}}}{r_{z_{o}}^{\alpha} \left(\pi \lambda\right)^{\frac{\alpha \eta}{2}}}\right)\!\right)\\ &\quad + R_{MQ}. \end{aligned}  $$

Here, *ζ*_*i*_ and *v*_*i*_ are the weights and nodes for the Gauss-Hermite quadrature of order *L*. Similarly, *κ*_*j*_ and *x*_*j*_ are the weights and nodes for Gauss-Hermite quadrature of order *M*. Finally, *β*_*q*_ and *δ*_*q*_ are the weight and nodes for the Gauss-Laguerre quadrature of order *Q*. Weights and nodes for Gauss-Laguerre quadrature of different orders are available in [36], Table (25.9), or can be calculated by a simple MATLAB program [33]. Terms *ε*_*L*_ and *R*_*MQ*_ are the errors of the approximations.

*Proof:* See Section 7.3.

### Scheme 2: Fractional compensation for the resultant effect of path loss and shadowing

When Scheme 2 is employed, SIR at the BS serving MS *z*_0_∈*Φ* can be written as

16 $$\begin{array}{*{20}l}  \text{SIR} = \frac{\rho r_{z_{0}}^{\alpha\left(\eta -1\right)} \hat{h}_{z_{0}}}{\sum_{z\in \Phi\backslash z_{0}} P_{z} r_{z}^{-\alpha} h_{z}}. \end{array} $$

Since power control partially inverts the effect of shadowing, $\hat {h}_{z_{0}}\sim \text {exp}(\mu)$, where *μ*∼lognormal(0,(1−*η*)*σ*). The coverage probability for this power control is given by the following theorem.

#### **Theorem 2**

In a single-tier Poisson cellular network with the closest BS cell association and fractional path loss and shadow inversion power control, the coverage probability can be given by

17 $$\begin{array}{*{20}l} {}P_{c}(T) &= 2\sqrt{\pi}\lambda \sum\limits_{i=1}^{L} \zeta_{i} \int_{0}^{\infty} r_{z_{0}} \text{exp}\left(-\pi \lambda r_{z_{0}}^{2}\right) \\ {}&\quad\times \mathcal{L}_{I_{\Phi\backslash z_{0}}}\!\left(s = \frac{T\ \text{exp}\left(-\sqrt{2} (1-\eta)\sigma v_{i}\right)}{\rho r_{z_{0}}^{\alpha (\eta-1)}}\right) dr_{z_{0}} \\ {}&\quad+ \epsilon_{L}, \end{array} $$

where

18 $$ \begin{aligned} \mathcal{L}_{I_{\Phi\backslash z_{0}}}(s) &= \text{exp}\left(\frac{-2 \lambda}{\alpha -2} \sum\limits_{k=1}^{N} w_{k} \sum\limits_{j=1}^{M}\kappa_{j} \sum\limits_{q=1}^{Q} \beta_{q}\right. \\ &\quad\times \frac{r_{z_{0}}^{2-\alpha} s \rho\ {exp}\left(\sqrt{2} \sigma x_{j}\right) \delta_{q}^{\frac{\alpha \eta}{2}}}{\left(\pi \lambda\right)^{\frac{\alpha \eta}{2}} {exp}\left(\sqrt{2} \xi u_{k} \eta\right)}\\ &\quad\left.\times \,\,{\!~\!}_{2}F_{1}\left(\!1, \frac{\alpha\,-\,2}{\alpha}, 2\,-\,\frac{2}{\alpha}, \!\frac{-s \rho\ \text{exp}\left(\sqrt{2}\sigma x_{j}\right)\delta_{q}^{\frac{\alpha \eta}{2}}}{r_{z_{0}}^{\alpha}\left(\pi \lambda\right)^{\frac{\alpha \eta}{2}}\text{exp}\left(\sqrt{2}\xi u_{k} \eta\right)}\!\right)\!\!\right) \\ &\quad+ R_{NMQ}. \end{aligned}  $$

Here, *ζ*_*i*_ and *v*_*i*_ are the weights and abscissas for the Gauss-Hermite quadrature of order *L*>1. Similarly, *w*_*k*_ and *u*_*k*_ are the weights and abscissas for the Gauss-Hermite quadrature of order *N*>1. *κ*_*j*_ and *x*_*j*_ are the weights and abscissas for Gauss-Hermite quadrature of order *M*>1. *β*_*q*_ and *δ*_*q*_ are the weight abscissas for the Gauss-Laguerre quadrature of order *Q*. Terms *ε*_*L*_ and *R*_*NMQ*_ are the errors of the approximations.

*Proof:* See Section 7.4.

### Scheme 3: Partial compensation for path loss and complete inversion of shadowing

In Scheme 3, path loss is partially compensated while the shadowing is completely inverted. Therefore, the SIR at BS serving MS *z*_0_∈*Φ* can be written as

19 $$  \text{SIR} = \frac{\rho r_{z_{0}}^{\alpha (\eta-1)} g_{z_{0}}}{\sum_{z\in \Phi\backslash z_{0}} P_{z} r_{z}^{-\eta} h_{z}},  $$

where *g*∼exp(1) is the power gain of the serving BS-MS channel due to Rayleigh multipath fading. The uplink coverage probability under this power control scheme is given by the following theorem.

#### **Theorem 3**

In a single-tier Poisson cellular network with the closest BS cell association and fractional path loss compensation and complete shadow inversion power control, the coverage probability is approximated by

20 $$\begin{array}{*{20}l}  P_{c} &= 2 \pi \lambda\int_{0}^{\infty}r_{z_{0}} \text{exp}\left(-\pi \lambda r_{z_{0}}^{2}\right) \\ &\quad \times \mathcal{L}_{I_{\Phi\backslash z_{0}}}\left(\frac{T r_{z_{0}}^{\alpha(1-\eta)}}{\rho}\right) dr_{z_{0}}, \end{array} $$

where,

21 $$ \begin{aligned} \mathcal{L}_{I_{\Phi\backslash z_{0}}}(s) &= \text{exp}\left(\frac{-2 \lambda}{\alpha -2} \sum\limits_{k=1}^{N} w_{k} \sum\limits_{j=1}^{M}\kappa_{j} \sum\limits_{q=1}^{Q} \beta_{q}\right. \\ &\quad\times \frac{r_{z_{0}}^{2-\alpha} s \rho\ \text{exp}\left(\sqrt{2} \sigma x_{j}\right) \delta_{q}^{\frac{\alpha \eta}{2}}}{\left(\pi \lambda\right)^{\frac{\alpha \eta}{2}} \text{exp}\left(\sqrt{2} \xi u_{k} \right)}\\ &\quad\left.\times \,\,{\!~\!}_{2}F_{1}\left(1, \frac{\alpha\,-\,2}{\alpha}, 2\,-\,\frac{2}{\alpha}, \frac{-s \rho\ \text{exp}\left(\sqrt{2}\sigma x_{j}\right)\delta_{q}^{\frac{\alpha \eta}{2}}}{r_{z_{0}}^{\alpha}\left(\pi \lambda\right)^{\frac{\alpha \eta}{2}}\text{exp}\left(\sqrt{2}\xi u_{k} \right)}\!\right)\!\right) \\ &\quad+ R_{NMQ}. \end{aligned}  $$

Here, *w*_*k*_ and *u*_*k*_ are the weights and abscissas for the Gauss-Hermite quadrature of order *N*>1. *κ*_*j*_ and *x*_*j*_ are the weights and abscissas for the Gauss-Hermite quadrature of order *M*>1. *β*_*q*_ and *δ*_*q*_ are the abscissas and weight factors for the Gauss-Laguerre quadrature of order *Q*>1. Term *R*_*NMQ*_ is the errors of the approximation.

*Proof:* See Section 7.5.

## Numerical results

This section presents numerical and simulation results and investigates the effect of power control factor, standard deviations of shadowing, and intensity of BSs on the uplink coverage probability. In the numerical derivations (Section 7), natural logarithm is used instead of logarithm with base 10. Therefore, PDFs (24) and (30) are scaled versions of the actual PDFs [2]. Thus, the standard deviations of shadowing in dB *σ*_dB_ and *ξ*_dB_ given in the following figures are *σ*_dB_=(10/ln10)*σ* dB and *ξ*_dB_=(10/ln10)*ξ* dB.

**Fig. 1:**
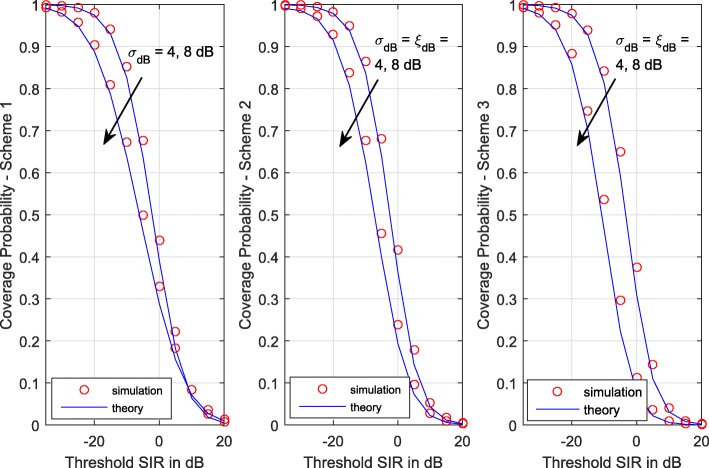
Coverage probability vs SIR threshold for the three power control schemes under different degrees of shadowing. *η*=0.5. *λ*=0.5 BSkm^−2^, *α*=3.5

In Fig. 1, we compare analytical coverage probability curves with the simulation results for three TPC schemes and different degrees of shadowing. We observe that our analytical expressions closely match the simulation results and accurately capture the performance trends. Further, lower *σ*_dB_ and *ξ*_dB_ (less severe shadowing) improve coverage. For analytical expressions, we use 30-points Hermite quadratures and 15-points Gauss-Laguerre quadratures.

**Fig. 2:**
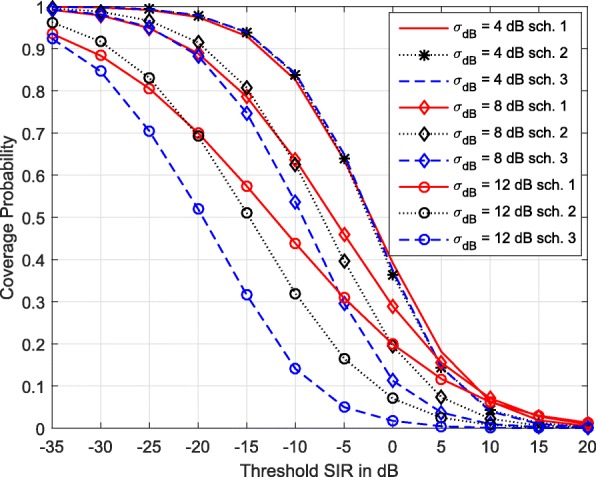
Comparison of coverage of the three power control schemes for different degrees of shadowing. *η*=0.5. *λ*=0.5 BSkm^−2^, *α*=3.5, *σ*_dB_=*ξ*_dB_

Figure 2 compares the coverage of the three TPC schemes under various degrees of shadowing. When shadowing is less severe, they achieve similar performance. For example, their coverage probabilities are more or less the same when *σ*_dB_=*ξ*_dB_=4 dB. This suggests that, in an environment with less severe shadowing, TPC using path loss inversion is sufficient. Consequently, frequent channel state measurements such that they capture the variations due to shadowing are not essential for proper power control. It is sufficient to capture channel state changes due to path loss. However, as the shadowing increases, the coverage probabilities differ significantly. At low SIR thresholds, compensating for the aggregate effect of path loss and shadowing (Scheme 2) improves coverage, especially for higher degrees of shadowing. However, at high SIR thresholds, path loss inversion (Scheme 1) provides better coverage compared to other two TPC schemes. Of these three, Scheme 3 results in the lowest coverage. This is because, although complete inversion of shadowing (Scheme 3) improves the received signal strength of the desired signal, it also increases the transmit powers of interfering MSs, resulting in higher aggregate interference power.

**Fig. 3:**
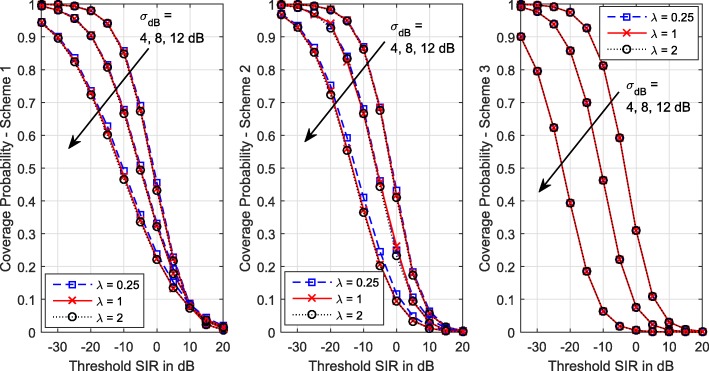
Coverage probability of the three power control schemes vs SIR threshold for different BS intensities and degrees of shadowing. *η*=0.5. *λ* is in BSs/km ^2^,*α*=3.5, *σ*_dB_=*ξ*_dB_

Figure 3 shows the coverage for different BS densities and shadowing levels. We see that the BS density has no impact on the coverage probabilities of Schemes 1 and 3, but severity of shadowing does. A similar observation can be made for Scheme 2, except for higher shadowing standard deviation values. For example, for $\tilde {\sigma } = \tilde {\xi } = 12$, coverage slightly lowers as the BS intensity increases. This is because increasing the intensity of BSs not only increases the intensity of co-channel interfering users but also increases the received signal power at the serving BSs by bringing in BSs closer to their users. Reference [8] has shown that with minimum path loss association and full PLI power control (Scheme 1 with *η*=1), uplink coverage is invariant of the BS density. Figure 3 corroborates this claim also for fractional power control (*η*<1) under Schemes 1 and 3 for all shadowing levels and for lower shadowing levels under Scheme 2.

**Fig. 4:**
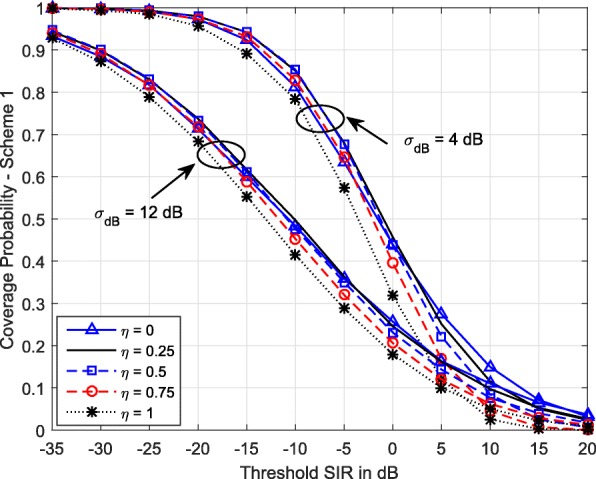
Variation of coverage of Scheme 1 vs SIR threshold for different *η* values. *λ*=0.5 BSs/km^2^, *α*=3.5

Figure 4 shows the coverage probability of Scheme 1 for different values of *η* and different degrees of shadowing. Clearly, the coverage is smallest when the path loss is completely compensated. This is because higher value of *η* helps the cell edge users meet their SIR target, but at the cost of higher interference level in the network. This also reduces the SINR experienced by cell center users. Therefore, the spatially averaged coverage probability is reduced. Therefore, careful choice of TPC parameters is essential for proper designing of a cellular network. We also observe that at high SIR threshold (*T*>0 dB), the *η*=0 (no power control; each MS transmits with the same transmit power regardless of the path loss) results in better coverage. Further, the variation of coverage with *η* is similar for the two shadowing severities considered.

**Fig. 5:**
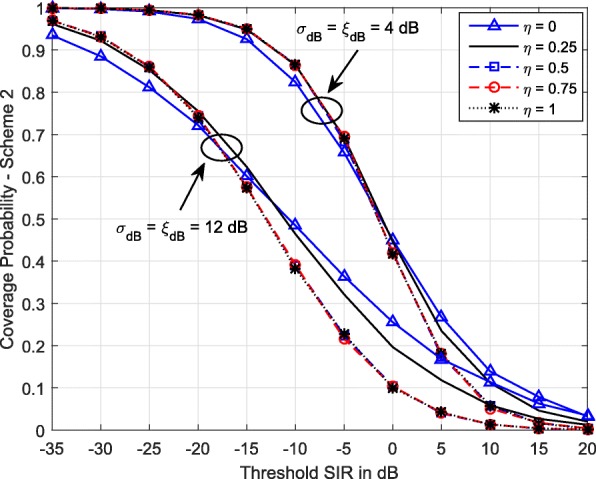
Coverage probability of Scheme 2 vs SIR threshold for different *η* values. *λ*=0.5 BSs/km^2^, *α*=3.5, *ρ*=−30 dBm

Figure 5 shows the coverage probability of Scheme 2 for various values of *η* and two levels of shadowing. Note that *η*=1 represents complete compensation for path loss and shadowing, resulting in a constant received power level *ρ* at the serving BS. *η*=0 represents no power control, resulting in each MS transmitting with constant power. At low SIR thresholds, complete compensation provides the highest coverage, while compensation for both path loss and shadowing (*η*>0) results in higher coverage compared to that of no power control (*η*=0). However, at high SIR thresholds, *η*=0 results higher coverage probability. Also, Fig. 5 shows that the performance gap widens, when the shadowing standard deviation increases. Therefore, we can conclude that at low SIR thresholds, complete elimination of shadow fading and path loss improves coverage, while at high SIR thresholds, power control reduces the coverage probability.

**Fig. 6:**
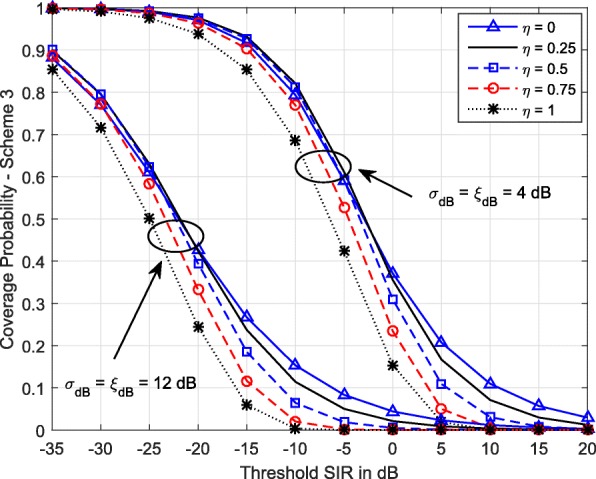
Coverage probability of Scheme 3 vs SIR threshold for different *η* values. *λ*=0.5 BSs/km^2^, *α*=3.5, *ρ*=−30 dBm

Figure 6 shows the variation of coverage probability with power control factor *η* for Scheme 3. When *η*=1, both path loss and shadowing are fully compensated for, resulting in a constant received power level *ρ* at the serving BS for all the MSs regardless of the path loss and shadowing they experience. On the other hand, *η*=0 only compensates for shadowing. This provides higher coverage probability for higher SIR thresholds, but not otherwise. Also, as the compensation factor increases above 0.5, coverage drops considerably. Therefore, we can conclude that at low SIR thresholds, complete elimination of shadowing and partial compensation of path loss gives better coverage, while at high SIR thresholds, inverting only the effect of shadowing improves coverage.

## Conclusions

Three uplink power control schemes for cellular networks with path loss and composite Rayleigh-lognormal fading have been investigated. They are fractional compensation of (1) path loss only, (2) the combined effect of path loss and shadowing, and (3) path loss and complete inversion of shadowing. Approximate PDF and CDF expressions have been derived for the transmit power and the coverage probabilities. The results have been validated via simulations.

This study leads to several observations. First, shadowing clearly has negative impact on network coverage. Second, at low SIRs, compensating for the aggregate effect of path loss and shadowing (Scheme 2) improves coverage compared to the other two schemes. However, at high SIRs, inverting only path loss (Scheme 1) provides the best coverage. Further, of the three, power control Scheme 3 gives the worst coverage. Third, previous research has observed that the BS intensity has little effect on the coverage, when minimum path loss association and full path loss inversion power control (Scheme 1 with *η*=1) is used in uplink. We find the same holds true for Schemes 1 and 3 for all shadowing levels and *η*<1. Further, it also holds for Scheme 2 under light shadowing. However, in all three schemes the extent to which path loss and shadowing are compensated has a significant effect on the coverage probability. Therefore, proper selection of uplink power control parameters is essential in cellular networks. Future research may encompass investigation of the effectiveness of power control schemes considered in this work in a heterogeneous cellular network setup, which includes different types of BSs and access points. In this setup, performance trends may be significantly different due to the use of different types of BSs with a range of capabilities and antenna heights, different cell association policies, and user offloading.

## Proofs of lemmas and theorems

### Proof of Lemma 2

When the aggregate effect of path loss and shadowing is partially compensated, transmit power *P*_*z*_ is given by (2). Using (2), the CDF of *P*_*z*_ can be written as

22 $$\begin{array}{*{20}l} F_{P_{z}}(t) &= \text{Pr} \left(\rho \left(r^{-\alpha} h_{zy}\right)^{-\eta} < t \right),\\ & = \mathcal{E}_{h_{zy}}\left[\text{Pr}\left(r < \left(\frac{t}{\rho}\right)^{\frac{1}{\eta \alpha}} h_{zy}^{\frac{1}{\alpha}}|h_{zy}\right) \right],\\ &\quad 0<t<\infty. \end{array} $$

Using the PDF of *r* given in (4), (22) can be written as

23 $$\begin{array}{*{20}l}  F_{P_{z}}(t) &= \mathcal{E}_{h_{zy}}\left[1-\text{exp}\left(-\pi \lambda \left(\frac{t}{\rho}\right)^{\frac{2}{\alpha \eta}}h_{zy}^{\frac{2}{\alpha}}\right) \right],\\ &\quad 0<t<\infty. \end{array} $$

The PDF of *h*_*zy*_ is given by

24 $$\begin{array}{*{20}l}  f_{h_{zy}}(h) &= \frac{1}{\sqrt{2 \pi}\xi h}\text{exp}\left(\frac{-\left(\text{ln}(h)\right)^{2}}{2\xi^{2}} \right),\ 0 <h<\infty. \end{array} $$

Substituting (24) in (23) and using the change of variable $u = \text {ln}(h)/\sqrt {2}\xi $,

25 $$\begin{array}{*{20}l}  F_{P_{z}}(t) &= 1-\frac{1}{\sqrt{\pi}}\int_{-\infty}^{\infty}\text{exp}\left(-u^{2}-\pi \lambda \left(\frac{t}{\rho}\right)^{\frac{2}{\alpha \eta}}\right. \\ &\quad \left.\times \text{exp}\left(\frac{2 \sqrt{2}\xi u}{\alpha}\right)\right) du,\ 0<t<\infty. \end{array} $$

Solving the integration in (25) by Gauss-Hermite quadrature, we obtain (8). PDF of *P*_*z*_ (9) is obtained by differentiating (8).

### Proof of Lemma 3

In this power control scheme, each MS transmits with power given by (3). The CDF of the transmit power of MS *z*∈*Φ*, *P*_*z*_, can be derived as follows.

26 $$\begin{array}{*{20}l}  F_{P_{z}(t)}&=\text{Pr}\left(\rho r^{\alpha \eta} h_{zy}^{-1}<t\right), \\ &=\mathcal{E}_{h_{zy}}\left[\text{Pr}\left(r < \left(\frac{h_{zy} t}{\rho}\right)^{\frac{1}{\alpha \eta}}|h_{zy}\right)\right]. \end{array} $$

Using the PDFs of *r* given by (4), (26) can be written as

27 $$\begin{array}{*{20}l}  F_{P_{z}(t)} &=\mathcal{E}_{h_{zy}}\left[1-\text{exp}\left(-\pi \lambda\left(\frac{h_{zy}t}{\rho}\right)^{2/\alpha\eta}\right)\right]. \end{array} $$

Substituting (24) in (27) and using the change of variable $u = \frac {\text {ln}(h)}{\sqrt {2}\xi }$,

28 $$\begin{array}{*{20}l}  F_{P_{z}}(t)&= 1 - \frac{1}{\sqrt{\pi}}\int_{-\infty}^{\infty}\text{exp}\left(-u^{2}-\pi \lambda\left(\frac{t}{\rho}\right)^{2/\alpha\eta}\right. \\ &\quad \left.\times \text{exp}\left(\frac{2\sqrt{2}\xi u}{\alpha \eta}\right)\right)du. \end{array} $$

Solving the integration in (28) by Gauss-Hermite quadrature, we obtain (10). Now, by differentiating (10), we can obtain (12).

### Proof of Theorem 1

For power control Scheme 1, SIR at the BS serving MS *z*_0_ is given by (13). Therefore, the coverage probability can be written as

29 $$\begin{array}{*{20}l}  P_{c} (T) &= \text{Pr}\left(\text{SIR} > T\right)=\text{Pr}\left(h_{z_{0}} > \frac{T I_{\Phi\backslash z_{0}}}{\rho r_{z_{0}}^{\alpha \left(\eta -1\right)}}\right), \end{array} $$

where $I_{\Phi \backslash z_{0}} = \sum _{z\in \Phi \backslash z_{0}} P_{z} r_{z}^{-\alpha } h_{z}$ is the total interference power from all the co-channel MSs. Since composite Rayleigh-lognormal fading is considered, $h_{z_{0}}\sim \text {exp}(\mu)$ where *μ*∼lognormal(0,*σ*). Now, the PDF of $h_{z_{0}}$ can be written as [2]

30 $$\begin{array}{*{20}l}  f_{h_{z_{0}}}(h) &= \int_{0}^{\infty} \frac{1}{\mu}\text{exp}\left(-\frac{h}{\mu}\right) \frac{1}{\sqrt{2 \pi}\sigma \mu}\\ &\ \ \ \ \times \text{exp}\left(\frac{-\left(\text{ln}(\mu)\right)^{2}}{2 \sigma^{2}}\right) d\mu. \end{array} $$

Substituting (30) in (29) introduces computational and analytical difficulties. To overcome these challenges, we use an approach similar to that proposed in [37]. Using change of variable $\text {ln}(h)/\sqrt {2}\sigma = v$, (30) can be written as

31 $$\begin{array}{*{20}l}  f_{h_{z_{0}}}(h)&=\frac{1}{\sqrt{\pi}}\int_{-\infty}^{\infty}\text{exp}\left(-\sqrt{2}\sigma v\right. \\ &\quad \left.- h\ \text{exp}\left(-\sqrt{2}\sigma v\right) - v^{2}\right) dv. \end{array} $$

(31) has the form of Gauss-Hermite integration, which can be approximated as

32 $$\begin{array}{*{20}l}  f_{h_{z_{0}}}(h) &= \sum_{i = 1}^{L} \frac{\zeta_{i}}{\sqrt{\pi}} \text{exp}\left(-\sqrt{2}\sigma v_{i} \right.\\ &\quad \left.- h\ \text{exp}\left(-\sqrt{2}\sigma v_{i}\right)\right) + O_{L}, \end{array} $$

where *ζ*_*i*_ and *v*_*i*_ are the weights and abscissas determined by Hermite polynomial after *L* is chosen. *L* represents the remainder terms that decrease to zero as *L* increases to infinity.

Using (32), the complementary cumulative distribution function (CCDF) of $h_{z_{0}}$ can be written as

33 $$\begin{array}{*{20}l}  {}\overline{F}_{h_{z_{0}}}(h) &= \sum_{i = 1}^{L} \frac{\zeta_{i}}{\sqrt{\pi}} \text{exp}\left(- h\ \text{exp}\left(-\sqrt{2} \sigma v_{i}\right)\right) + O'_{L}, \end{array} $$

where $O^{\prime }_{L}$ is the error in the approximation due to *O*_*L*_ in (32). Substituting (33) in (29), *P*_*c*_(*T*) can be written as

34 $$ \begin{aligned} P_{c} (T) &= \sum_{i = 1}^{L} \frac{\zeta_{i}}{\sqrt{\pi}} \\ &\quad \times \mathcal{E}_{r_{z_{0}}}\!\! \left[ \mathcal{E}_{I_{\Phi\backslash z_{0}}}\left[\text{exp}\left(\frac{-I_{\Phi\backslash z_{0}} T\ \text{exp}\left(-\sqrt{2} \sigma v_{i}\right)}{\rho r_{z_{0}}^{\alpha (\eta-1)}}\right)\right]\right] \\ &\quad + \epsilon_{L}, \end{aligned}  $$

where *ε*_*L*_ is the error in the approximation due to $O^{\prime }_{L}$ of (33). Using the definition of the Laplace transform, (34) can be written as

35 $$\begin{array}{*{20}l}  {}P_{c} (T) &= \sum_{i = 1}^{L} \frac{\zeta_{i}}{\sqrt{\pi}} \\ {}&\quad \times \mathcal{E}_{r_{z_{0}}}\left[\mathcal{L}_{I_{\Phi\backslash z_{0}}}\left(s = \frac{T\ \text{exp}\left(-\sqrt{2} \sigma v_{i}\right)}{\rho r_{z_{0}}^{\alpha (\eta-1)}}\right)\right] + \epsilon_{L}, \end{array} $$

where $\mathcal {L}_{I_{\Phi \backslash z_{0}}}(s)$ is the Laplace transform of the PDF of random variable $I_{\Phi \backslash z_{0}}$. Substituting the PDF of $r_{z_{0}}$ given by (4) in (35), we obtain (14). $\mathcal {L}_{I_{\Phi \backslash z_{0}}}(s)$ can be derived as follows.

36 $$\begin{array}{*{20}l}  \mathcal{L}_{I_{\Phi\backslash z_{0}}}(s) &= \mathcal{E}_{\Phi, h_{z}, P_{z}}\left[\text{exp}\left(-s \sum_{z\in \Phi\backslash z_{0}}P_{z} r_{z}^{-\alpha} h_{z}\right)\right]\\ &=\mathcal{E}_{\Phi, h_{z}, P_{z}}\left[\prod_{z\in \Phi\backslash z_{0}}\text{exp}\left(-sP_{z} r_{z}^{-\alpha} h_{z}\right)\right],\\ &\stackrel{a}{=}\text{exp}\left(-2 \pi \lambda\int_{r_{z_{0}}}^{\infty}r_{z}\right.\\ &\quad \left.\times \mathcal{E}_{h_{z},P_{z}}\left[1-\text{exp}\left(-s P_{z} r_{z}^{-\alpha} h_{z}\right)\!\right] dr_{z}\vphantom{\int_{r_{z_{0}}}^{\infty}}\right), \end{array} $$

where step (a) follows from the definition of probability generating functional of PPP [32]. Consider $\mathcal {I}= \mathcal {E}_{h_{z}, P_{z}}$$\left [1-\text {exp}\left (-s P_{z} r_{z}^{-\alpha } h_{z}\right)\right ]$. Using the PDF of *h*_*z*_ given by (32), $\mathcal {I}$ can be written as

37 $$\begin{array}{*{20}l}  \mathcal{I}&= \mathcal{E}_{P_{z}} \left[ 1-\sum_{j=1}^{M} \frac{\kappa_{j}}{\sqrt{\pi}} \text{exp}\left(-\sqrt{2} \sigma x_{j} \right)\right. \\ &\quad \left.\times \int_{0}^{\infty}\!\! \text{exp}\left(\! -h_{z} \left(\text{exp}\left(\! -\sqrt{2}\sigma x_{j}\right) + s P_{z} r_{z}^{-\alpha}\right)\right) dh_{z} \right]\\ &\quad + O_{M},\\ &= \mathcal{E}_{P_{z}} \left[ 1-\sum_{j=1}^{M} \frac{\kappa_{j}}{\sqrt{\pi}}\frac{1}{1 + s P_{z} r_{z}^{-\alpha}\text{exp}\left(\sqrt{2} \sigma x_{j}\right)} \right] \\ &\quad + O_{M}, \end{array} $$

where *O*_*M*_ is the error due to using the approximate expression (32). Substituting (6) in (37) and using the fact that $\sum _{j=1}^{M} \kappa _{j} = \sqrt {\pi }$,

38 $$\begin{array}{*{20}l}  \mathcal{I}&= \frac{2 \sqrt{\pi} \lambda}{\alpha \eta \rho^{\frac{2}{\alpha \eta}}} \sum_{j=1}^{M} \kappa_{j} \int_{0}^{\infty}\frac{P_{z}^{\frac{2}{\alpha \eta}-1}}{1 + \frac{r_{z}^{\alpha}}{s P_{z} \text{exp}\left(\sqrt{2}\sigma x_{j}\right)}} \\ &\quad \times \text{exp}\left(-\pi \lambda \left(\frac{P_{z}}{\rho}\right)^{\frac{2}{\alpha \eta}}\right) dP_{z} + O_{M}. \end{array} $$

Using the change of variable $\delta = \pi \lambda \left (\frac {P_{z}}{\rho }\right)^{\frac {2}{\alpha \eta }}$, (38) can be written as

39 $$\begin{array}{*{20}l}  \mathcal{I}&= \sum_{j=1}^{M} \frac{\kappa_{j}}{\sqrt{\pi}} \int_{0}^{\infty} \frac{\text{exp}(-\delta)}{1 + \frac{r_{z}^{\alpha} \left(\pi \lambda\right)^{\frac{\alpha \eta}{2}}}{s \rho\ \text{exp}\left(\sqrt{2}\sigma x_{j}\right) \delta^{\frac{\alpha \eta}{2}}}} dy + O_{M}. \end{array} $$

(39) can be approximated as a Gauss-Laguerre quadrature sum as given below.

40 $$\begin{array}{*{20}l}  \mathcal{I}&= \sum_{j=1}^{M} \frac{\kappa_{j}}{\sqrt{\pi}} \sum_{q = 1}^{Q} \beta_{q} \frac{1}{1 + \frac{r_{z}^{\alpha} \left(\pi \lambda\right)^{\frac{\alpha \eta}{2}}}{s \rho\ \text{exp}\left(\sqrt{2}\sigma x_{j}\right) \delta_{q}^{\frac{\alpha \eta}{2}}}} + O_{MQ}, \end{array} $$

where *β*_*q*_ and *δ*_*q*_ are the abscissas and weight factors for the Gauss-Laguerre integration [36], Table (25.9), and *O*_*MQ*_ is the error in the approximation. Substituting (40) in (36),

41 $$\begin{array}{*{20}l}  \mathcal{L}_{I_{\Phi\backslash z_{0}}}(s) & = \text{exp} \left(\vphantom{\int_{r_{z_{0}}}^{\infty}\frac{r_{z}}{1 + \frac{r_{z}^{\alpha} \left(\pi \lambda\right)^{\frac{\alpha \eta}{2}}}{s \rho\ \text{exp}\left(\sqrt{2}\sigma x_{j}\right) \delta_{q}^{\frac{\alpha \eta}{2}}}}}-2 \sqrt{\pi} \lambda \sum_{j=1}^{M} \kappa_{j} \sum_{q = 1}^{Q} \beta_{q}\right. \\ &\quad \left. \times \int_{r_{z_{0}}}^{\infty}\frac{r_{z}}{1 + \frac{r_{z}^{\alpha} \left(\pi \lambda\right)^{\frac{\alpha \eta}{2}}}{s \rho\ \text{exp}\left(\sqrt{2}\sigma x_{j}\right) \delta_{q}^{\frac{\alpha \eta}{2}}}} d_{r_{z}}\right) + R_{MQ}, \end{array} $$

where *R*_*MQ*_ is the error in the approximation. Solving (41), we obtain (15) of Theorem 1.

### Proof of Theorem 2

When power control Scheme 2 is employed, SIR at the BS serving MS *z*_0_∈*Φ* is given by (16). Therefore, the coverage probability can be written as

42 $$\begin{array}{*{20}l}  P_{c}(T) &= \text{Pr}\left(\hat{h}_{z_{0}} > \frac{T I_{\Phi\backslash z_{0}}}{\rho r_{z_{0}}^{\alpha(\eta-1)}}\right). \end{array} $$

The CCDF of $\hat {h}_{z_{0}}$ can be obtained by replacing *σ* by (1−*η*)*σ* in (33). Following approach similar to the derivation of (35), (42) can be written as

43 $$\begin{array}{*{20}l} {}P_{c} (T) &= \sum_{i = 1}^{L} \frac{\zeta_{i}}{\sqrt{\pi}}\\ {}&\quad \times \mathcal{E}_{r_{z_{0}}}\!\left[\mathcal{L}_{I_{\Phi\backslash z_{0}}}\!\left(s = \frac{T\ \text{exp}\left(-\sqrt{2} (1-\eta)\sigma v_{i}\right)}{\rho r_{z_{0}}^{\alpha (\eta-1)}}\right)\right] \\ {}&\quad + \epsilon_{L}, \end{array} $$

where *ε*_*L*_ is the error in the approximation. Substituting (4) in (43), we obtain (17). Following steps similar to the derivation of (36), $\mathcal {L}_{I_{\Phi \backslash z_{0}}}(s)$ for power control Scheme 2 can be derived as follows.

44 $$\begin{array}{*{20}l}  \mathcal{L}_{I_{\Phi\backslash z_{0}}}(s) &= \text{exp}\left(-2 \pi \lambda \int_{r_{z_{0}}}^{\infty}r_{z}\right. \\ &\quad \left. \times \left(1-\mathcal{E}_{P_{z}, h_{z}}\left[\text{exp}\left(-s P_{z} r_{z}^{-\alpha} h_{z}\right)\right]\right) dr_{z}\vphantom{\int_{r_{z_{0}}}^{\infty}}\right). \end{array} $$

Consider $\mathcal {B}=1-\mathcal {E}_{P_{z}, h_{z}}\left [\text {exp}\left (-s P_{z} r_{z}^{-\alpha } h_{z}\right)\right ]$. $\mathcal {B}$ is similar to $\mathcal {I}$ given in (37), but with the PDF of *P*_*z*_ given by (9). Therefore, $\mathcal {B}$ can be written as

45 $$\begin{array}{*{20}l} \mathcal{B}&= \frac{2\lambda}{\alpha \eta \rho^{\frac{2}{\alpha \eta}}} \sum_{k=1}^{N} w_{k} \sum_{j=1}^{M}\kappa_{j} \text{exp}\left(\frac{2\sqrt{2} \xi u_{k}}{\alpha}\right)\\ &\quad \times \int_{0}^{\infty}\frac{P_{z}^{\frac{2}{\alpha \eta}-1}}{1 + \frac{r_{z}^{\alpha}}{s P_{z} \ \text{exp}\left(\sqrt{2}\sigma x_{j}\right)}} \\ &\quad \times \text{exp}\left(-\pi \lambda \left(\frac{P_{z}}{\rho}\right)^{\frac{2}{\alpha \eta}} \text{exp}\left(\frac{2\sqrt{2} \xi u_{k}}{\alpha}\right)\right) dP_{z} \\ &\quad + O_{NM}. \end{array} $$

Here, *O*_*NM*_ is the error term due to *O*_*M*_. Using the change of variable $\delta = \pi \lambda \left (\frac {P_{z}}{\rho }\right)^{\frac {2}{\alpha \eta }} \text {exp}\left (\frac {2\sqrt {2} \xi u_{k}}{\alpha }\right)$

46 $$\begin{array}{*{20}l}  \mathcal{B}&\,=\, \frac{1}{\pi} \sum_{k=1}^{N} w_{k} \sum_{j=1}^{M}\kappa_{j}\!\! \int_{0}^{\infty}\!\! \!\!\!\frac{e^{-\delta}}{1 + \frac{r_{z}^{\alpha} \left(\pi\lambda\right)^{\frac{\alpha\eta}{2}}\text{exp}\left(\sqrt{2} \xi u_{k} \eta\right)}{s \rho\ \text{exp}\left(\sqrt{2}\sigma x_{j}\right) \delta^{\frac{\alpha \eta}{2}}}}d\delta\! \\ &\quad +\! O_{NM}. \end{array} $$

Solving the integral in (46) by Gauss-Laguerre quadrature,

47 $$\begin{array}{*{20}l}  \mathcal{B}&\,=\,\frac{1}{\pi}\!\sum_{k=1}^{N} w_{k} \sum_{j=1}^{M}\kappa_{j} \sum_{q=1}^{Q} \beta_{q} \frac{1}{1\,+\,\frac{r_{z}^{\alpha} \left(\pi\lambda\right)^{\frac{\alpha\eta}{2}}\text{exp}\left(\sqrt{2} \xi u_{k} \eta\right)}{s \rho\ \text{exp}\left(\sqrt{2}\sigma x_{j}\right) \delta_{q}^{\frac{\alpha \eta}{2}}}}\!\\ &\quad +\! O_{NMQ}. \end{array} $$

Here, *β*_*q*_ and *δ*_*q*_ are the abscissas and weight factors for the Gauss-Laguerre quadrature of order *Q*>1. *O*_*NMQ*_ is the error in the approximation. Substituting (47) in (44) and solving the integration, we obtain (18) of Theorem 2.

### Proof of Theorem 3

When power control Scheme 3 is employed, SIR at the BS serving MS *z*_0_∈*Φ* is given by (19). Therefore, the coverage probability can be written as

48 $$\begin{array}{*{20}l}  P_{c}(T) &= \text{Pr}\left[g_{z_{0}} > \frac{T I_{\Phi\backslash {z_{0}}}}{\rho r_{z_{0}}^{\alpha (\eta-1)}}\right]\\ &\stackrel{a}{=}2 \pi \lambda \int_{0}^{\infty} \!\!\!r_{z_{0}} \text{exp}\left(-\pi \lambda r_{z_{0}}^{2}\right) \\ &\quad \times \mathcal{E}_{I_{\Phi\backslash z_{0}}}\left[\text{exp}\left(-\frac{I_{\Phi\backslash z_{0}} T r_{z_{0}}^{\alpha(1-\eta)}}{\rho}\right)\right] dr_{z_{0}}. \end{array} $$

In (48), step (a) follows due to *g*∼exp(1) and using the PDF of $r_{z_{0}}$ given in (4). Using the definition of the Laplace transform, *P*_*c*_(*T*) for power control Scheme 3 can be written as

49 $$\begin{array}{*{20}l}  P_{c} &= 2 \pi \lambda\int_{0}^{\infty}r_{z_{0}} \text{exp}\left(-\pi \lambda r_{z_{0}}^{2}\right) \\ &\quad \times \mathcal{L}_{I_{\Phi\backslash z_{0}}}\left(\frac{T r_{z_{0}}^{\alpha(1-\eta)}}{\rho}\right) dr_{z_{0}}, \end{array} $$

where $\mathcal {L}_{I_{\Phi \backslash {z_{0}}}}(s)$ is the Laplace transform of the PDF of aggregate interference $I_{\Phi \backslash {z_{0}}}$. This concludes the derivation of (20) of Theorem 3. Using steps similar to the derivation of (36), $\mathcal {L}_{I_{\Phi \backslash {z_{0}}}}(s)$ can be written as

50 $$\begin{array}{*{20}l} {}\mathcal{L}_{I_{\Phi\backslash z_{0}}}(s) &= \text{exp}\left(\vphantom{\int_{r_{z_{0}}}^{\infty}}-2 \pi \lambda\right. \\ &\quad \left. \times \int_{r_{z_{0}}}^{\infty}\left(1 - \mathcal{E}_{P_{z}, h_{z}} \left[\text{exp}\left(-s P_{z} r_{z}^{-\alpha} h_{z}\right)\right]\right)\right)r_{z} dr_{z}. \end{array} $$

Consider $\mathcal {A} = 1 - \mathcal {E}_{P_{z}, h_{z}} \left.\left [\text {exp}\left (-s P_{z} r_{z}^{-\alpha } h_{z}\right)\right ]\right)$. $\mathcal {A}$ is similar to $\mathcal {I}$ given in (37), but with the PDF of *P*_*z*_ given by (12). Therefore, $\mathcal {A}$ can be written as

51 $$\begin{array}{*{20}l} \mathcal{A}&= \frac{2 \lambda}{\rho^{\frac{2}{\alpha \eta}}\alpha \eta} \sum_{k=1}^{N} w_{k} \sum_{j=1}^{M}\kappa_{j}\int_{0}^{\infty} \frac{P_{z}^{\frac{2}{\alpha \eta}-1}}{1 + \frac{r_{z}^{\alpha}}{s P_{z} \text{exp}(\sqrt{2}\sigma x_{j})}} \\ &\quad \times\text{exp}\left(\frac{2 \sqrt{2}\xi u_{k}}{\alpha \eta}-\frac{\pi \lambda P_{z}^{\frac{2}{\alpha \eta}} \text{exp}\left(\frac{2 \sqrt{2} \xi u_{k}}{\alpha \eta}\right)}{\rho^{\frac{2}{\alpha \eta}}}\right)\!dP_{z} \\ &\quad + O_{NM}, \end{array} $$

where *O*_*NM*_ is the error in the approximation. Using the change of variable $\delta =\pi \lambda P_{z}^{\frac {2}{\alpha \eta }} \text {exp}\left (\frac {2 \sqrt {2} \sigma u_{i}}{\alpha \eta }\right) \rho ^{\frac {-2}{\alpha \eta }}$,

52 $$\begin{array}{*{20}l}  \mathcal{A} &= \frac{1}{\pi} \sum_{k=1}^{N} w_{k} \sum_{j=1}^{M}\kappa_{j} \\ &\quad \times \int_{0}^{\infty}\!\!\! \frac{\text{exp}(-\delta)}{1 + \frac{r_{z}^{\alpha}\left(\pi \lambda\right)^{\frac{\alpha \eta}{2}}\text{exp}(\sqrt{2} \sigma u_{k})}{s \rho\ \text{exp}(\sqrt{2}\xi x_{j}) \delta^{\frac{\alpha \eta}{2}}}} d\delta + O_{NM}. \end{array} $$

The integral in (52) can be approximated as a Gauss-Laguerre quadrature sum as given below.

53 $$\begin{array}{*{20}l}  \mathcal{A} &\,=\, \frac{1}{\pi}\!\sum_{k=1}^{N} w_{k} \sum_{j=1}^{M}\kappa_{j} \sum_{q = 1}^{Q}\beta_{q} \frac{1}{\!1\! + \frac{r_{z}^{\alpha}\left(\pi \lambda\right)^{\frac{\alpha \eta}{2}}\text{exp}(\sqrt{2} \sigma u_{k})}{s \rho\ \text{exp}(\sqrt{2}\xi x_{j}) \delta_{q}^{\frac{\alpha \eta}{2}}}}\!\\ &\quad +\!O_{NMQ}, \end{array} $$

where *O*_*NMQ*_ is the error term due to *O*_*NM*_ and representing integration in (52) as a Gauss-Laguerre quadrature sum. Substituting (53) in (50), we obtain (21) of Theorem 3.

## Footnotes

1 With a slight abuse of notation, we will use *z* to denote both the location of MS and MS itself, similarly for BS *y*.
